# Viable adhered *Staphylococcus aureus* highly reduced on novel antimicrobial sutures using chlorhexidine and octenidine to avoid surgical site infection (SSI)

**DOI:** 10.1371/journal.pone.0190912

**Published:** 2018-01-09

**Authors:** Andreas Obermeier, Jochen Schneider, Norbert Harrasser, Jutta Tübel, Heinrich Mühlhofer, Dominik Pförringer, Constantin von Deimling, Peter Foehr, Barbara Kiefel, Christina Krämer, Axel Stemberger, Matthias Schieker, Rainer Burgkart, Rüdiger von Eisenhart-Rothe

**Affiliations:** 1 Klinik für Orthopädie und Sportorthopädie, Klinikum rechts der Isar der Technischen Universität München, München, Germany; 2 II. Medizinische Klinik und Poliklinik, Klinikum rechts der Isar der Technischen Universität München, München, Germany; 3 Klinik und Poliklinik für Unfallchirurgie, Klinikum rechts der Isar der Technischen Universität München, München, Germany; 4 Klinik für Chirurgie, Experimentelle Chirurgie und Regenerative Medizin, Klinikum der Universität München, München, Germany; Royal College of Surgeons in Ireland, IRELAND

## Abstract

**Background:**

Surgical sutures can promote migration of bacteria and thus start infections. Antiseptic coating of sutures may inhibit proliferation of adhered bacteria and avoid such complications.

**Objectives:**

This study investigated the inhibition of viable adhering bacteria on novel antimicrobially coated surgical sutures using chlorhexidine or octenidine, a critical factor for proliferation at the onset of local infections. The medical need, a rapid eradication of bacteria in wounds, can be fulfilled by a high antimicrobial efficacy during the first days after wound closure.

**Methods:**

As a pretesting on antibacterial efficacy against relevant bacterial pathogens a zone of inhibition assay was conducted with middle ranged concentrated suture coatings (22 μg/cm). For further investigation of adhering bacteria in detail the most clinically relevant *Staphylococcus aureus* (ATCC^®^49230™) was used. Absorbable braided sutures were coated with chlorhexidine-laurate, chlorhexidine-palmitate, octenidine-laurate, and octenidine-palmitate. Each coating type resulted in 11, 22, or 33 μg/cm drug content on sutures. Scanning electron microscopy (SEM) was performed once to inspect the coating quality and twice to investigate if bacteria have colonized on sutures. Adhesion experiments were assessed by exposing coated sutures to *S*. *aureus* suspensions for 3 h at 37°C. Subsequently, sutures were sonicated and the number of viable bacteria released from the suture surface was determined. Furthermore, the number of viable planktonic bacteria was measured in suspensions containing antimicrobial sutures. Commercially available sutures without drugs (Vicryl^®^, PGA Resorba^®^, and Gunze PGA), as well as triclosan-containing Vicryl^®^ Plus were used as control groups.

**Results:**

Zone of inhibition assay documented a multispecies efficacy of novel coated sutures against tested bacterial strains, comparable to most relevant *S*. *aureus* over 48 hours. SEM pictures demonstrated uniform layers on coated sutures with higher roughness for palmitate coatings and sustaining integrity of coated sutures. Adherent *S*. *aureus* were found via SEM on all types of investigated sutures. The novel antimicrobial sutures showed significantly less viable adhered *S*. *aureus* bacteria (up to 6.1 log) compared to Vicryl^®^ Plus (0.5 log). Within 11 μg/cm drug-containing sutures, octenidine-palmitate (OL11) showed the highest number of viable adhered *S*. *aureus* (0.5 log), similar to Vicryl^®^ Plus. Chlorhexidine-laurate (CL11) showed the lowest number of *S*. *aureus* on sutures (1.7 log), a 1.2 log greater reduction. In addition, planktonic *S*. *aureus* in suspensions were highly inhibited by CL11 (0.9 log) represents a 0.6 log greater reduction compared to Vicryl^®^ Plus (0.3 log).

**Conclusions:**

Novel antimicrobial sutures can potentially limit surgical site infections caused by multiple pathogenic bacterial species. Therefore, a potential inhibition of multispecies biofilm formation is assumed. In detail tested with *S*. *aureus*, the chlorhexidine-laurate coating (CL11) best meets the medical requirements for a fast bacterial eradication. This suture coating shows the lowest survival rate of adhering as well as planktonic bacteria, a high drug release during the first–clinically most relevant– 48 hours, as well as biocompatibility. Thus, CL11 coatings should be recommended for prophylactic antimicrobial sutures as an optimal surgical supplement to reduce wound infections. However, animal and clinical investigations are important to prove safety and efficacy for future applications.

## Introduction

Surgical site infection (SSI) rates vary in the range of 2% to 20% depending on the chosen type of surgical procedure [1–4]. SSI generally poses a risk for patients due to an increased morbidity and even mortality [4]. Affected patients often need further surgical intervention leading to higher cost for the health care system [1, 5]. Several factors are involved in the onset of SSI, one of which is the surgical suture itself. The presence of foreign material highly reduces the critical number of bacteria facilitating a clinically relevant infection [6–8]. Furthermore, the capillarity of sutures supports the path of bacteria into wounds by soaked fluids. This so-called “wicking effect” triggers such infections. [9] Especially, the type of material and structure of surface determine the ability of bacteria to adhere and induce infections [9]. In this context, the number of viable adhered bacteria is considered an essential trigger for SSI related to suture material. The main issues are proliferation of attached bacteria and formation of persistent biofilms [9–11]. Once a biofilm has developed, it protects bacteria against the host’s immune system as well as systemically [12, 13] and locally applied antibiotics.

A possible solution to prevent suture-associated site infections is the use of antimicrobially coated sutures. These sutures can be used to inhibit viable adhered microbes and thus prevent biofilm formation. Clinical indications for antimicrobial sutures may be infection prophylaxis in susceptible patients (e.g. immunosuppression) and especially in surgical procedures with elevated risk of infection (e.g. contaminated surgical site). To our knowledge, so-called “Plus” sutures containing triclosan are the only antimicrobial sutures currently available on the European market.

A systematic literature review on antimicrobial sutures by Chang et al. identified seven randomized clinical trials finding no significant reduction of local infections by means of these materials [14]. However, these studies did not fulfill the recommended standards for meta-analyses [15]. In contrast, the latest independent meta-analyses indicate a beneficial use of antimicrobial sutures for wound closure containing triclosan [16, 17]. Due to this data, antimicrobial sutures are highly recommended as a supplementary step to reduce the risk of SSI [15]. Further studies showed high efficacy and cost reduction of antimicrobial sutures for infection prevention [18–22] and could clarify which indication benefits most from the use of available antimicrobial sutures [23, 24].

Apart from promising study results of triclosan-coated sutures, triclosan also has drawbacks including formation of toxic side products (e.g. chlorinated phenols, methyl triclosan) [25] and antibiotic resistances [26, 27], likely due to its prevalence in cosmetics and soap products [28, 29]. Additionally, triclosan promotes the protein mediated binding of staphylococci to host cells with the consequence of an increased number of nasal infections caused by *S*. *aureus* colonization in the presence of triclosan [30]. Due to these restrictions in the use of triclosan, alternatives are urgently needed. Chlorhexidine and octenidine are highly effective alternatives, inhibiting relevant pathogens of wound infections. Both antiseptics have a broad antibacterial spectrum as well as high biocompatibility indices [31–33]. Chlorhexidine is routinely used in oral surgery [31]. In combination with silver, chlorhexidine is also used for the antimicrobial protection of hernia meshes. These chlorhexidine meshes show antibacterial efficacy, safety and high tissue integration [34]. Octenidine is a clinically well-established skin and wound antiseptic solution and does not seem to select for resistance [35].

Chlorhexidine and octenidine have similar mechanism of action: Positively charged drug molecules bind to negative charges on bacterial cell walls, leading to membrane leakages and finally cell death [36, 37]. Both antiseptics are effective against the most gram-negative and gram-positive bacteria [37, 38], including the most clinically relevant pathogen genus staphylococci, causing wound and nosocomial infections [39, 40]. In order to support wound healing, a fast and if possible complete eradication of bacteria inside wounds after surgery is necessary. Therefore, administration of antimicrobial agents is recommended at high dosages and short time periods for prophylaxis to avoid formation of resistant bacteria [41, 42].

Antimicrobial sutures must fulfill a balancing act between inhibiting bacteria and sustaining biocompatibility to the healing wound consisting of eukaryotic tissue. In former studies, we adjusted the drug concentration dependent on efficacy and biocompatibility of novel antimicrobial suture coatings containing chlorhexidine diacetate [43] or octenidine dihydrochloride [44]. These studies used coatings based on fatty acid carriers to achieve delayed drug release systems and to sustain bacterial inhibition zones *in vitro*.

The aim of the present study was to investigate the effectiveness of novel chlorhexidine- or octenidine-coated sutures against adherent bacteria. At first, a zone of inhibition assay was conducted to determine the efficacy against several relevant pathogenic bacteria. Then, in order to investigate the effects of novel antimicrobially coated sutures on viable adhering bacteria in detail the clinically most relevant *S*. *aureus* was used. Therefore, coated suture samples were exposed to *S*. *aureus* suspensions. Scanning electron microscopy (SEM) was performed to inspect suture coatings before and adherent bacteria after *S*. *aureus* exposure. The viability of bacteria adhered on sutures was investigated after sonication. In addition, the viability of planktonic bacteria in the surrounding of coated sutures was measured. The tested novel coated sutures were compared to commercially available absorbable sutures without any drug cover as well as triclosan-containing Vicryl^®^ Plus sutures.

## Materials and methods

### Surgical sutures

In this study, uncoated braided absorbable—polyglycolic acid—suture Gunze (G: Gunze PGA, Kyoto, Japan) of 0.4 mm in diameter, corresponding to the United States Pharmacopeia standard USP1, was used to produce antimicrobial sutures by coating. Suture controls were commercial PGA Resorba® (R: Resorba, Nürnberg, Germany), Vicryl® and triclosan-containing Vicryl® Plus (V and VP, respectively: Ethicon, Norderstedt, Germany). Furthermore, Gunze PGA sutures only coated with fatty acids—palmitic acid (PA80) and lauric acid (LA80)—were tested to investigate potential effects of drug carriers only.

### Antimicrobial suture preparation using chlorhexidine and octenidine in fatty acid carriers

The formulation of coating solutions and the reproducibility of the dip coating process for antimicrobial coating of absorbable PGA sutures (Gunze) was described earlier in one of our studies for chlorhexidine diacetate [43] and octenidine dihydrochloride [44] based on fatty acids as drug carriers. These coating procedures resulted in an average coating weight of 2.2 mg ± 0.2 mg (n = 10) for 40 cm braided, absorbable sutures (USP1) [43, 44].

In the present study, four coating types were compared: Chlorhexidine in lauric acid (CL) or palmitic acid (CP) and octenidine in lauric acid (OL) or palmitic acid (OP). For each type of suture coating, three different solutions with defined concentrations of active agents were formulated. To obtain preparation solutions, antiseptic drugs and fatty acid carriers (palmitate or laurate) were dissolved in 99.8% ethanol with a total mass content of 5% (w/w). Sutures were dipped in these sterile coating solutions for 2 min, followed by a drying period of 2 hours. Then, the weight of coatings on sutures was measured via a precision balance (Atilon ATL-224, Acculab, Bradford, USA) and the resulting drug concentration per unit of length was calculated. This procedure generates antimicrobial sutures with 11 μg/cm, 22 μg/cm and 33 μg/cm for both chlorhexidine- and octenidine-containing sutures. An overview of the tested novel antimicrobial sutures and their coating composition for this study is given in Table 1.

**Table 1:** Overview of the prepared novel antimicrobially coated sutures.

A) chlorhexidine-coated sutures			B) octenidine-coated sutures			C) fatty acid carrier	
types of chlorhexidine coating		drug content (μg/cm)	types of octenidine coating		drug content (μg/cm)	content (μg/cm)	ratio (%)
chlorhexidine-laurate	CL11	11	octenidine-laurate	OL11	11	44	80
chlorhexidine-palmitate	CP11		octenidine-palmitate	OP11			
chlorhexidine-laurate	CL22	22	octenidine-laurate	OL22	22	33	60
chlorhexidine-palmitate	CP22		octenidine-palmitate	OP22			
chlorhexidine-laurate	CL33	33	octenidine-laurate	OL33	33	22	40
chlorhexidine-palmitate	CP33		octenidine-palmitate	OP33			

Chlorhexidine-coated sutures (A) and octenidine-coated sutures (B) and their coating compositions are shown in detail. For both types of sutures, the amount of antimicrobial substance per length of sutures after preparation resulting from a mean coating weight of 40 cm suture samples at 2.2 ± 0.2 mg (n = 7) is given. Additionally, the fatty acid content and ratio (C) is referred to the total weight of coating mass per cm length of the sutures.

In comparison to our chlorhexidine- or octenidine-containing antimicrobial sutures, the Vicryl® Plus control group suture contains 2.7 μg/cm triclosan within the European Union [29].

### Antimicrobial efficacy against multiple relevant pathogenic bacteria

In order to achieve information about a multispecies efficacy *Staphylococcus aureus* (ATCC^®^49230^™^), a methicillin-resistant *S*. *aureus* strain—short *MRSA* (ATCC^®^43300^™^), *Staphylococcus epidermidis* (ATCC^®^35984^™^), *Enterococcus faecalis* (ATCC^®^29212^™^) and *Escherichia coli* (ATCC^®^25922^™^) was used for general antibacterial suture tests. The zone of inhibition assay was conducted over a period of 48 hours to compare the species-dependent efficacy of each suture type using the middle ranged drug concentration in the amount of 22 μg/cm. Therefore, coated suture samples were placed on bacterial lawns on Agar plates (Mueller Hinton II), inoculated with a bacterial suspension at an optical density of 0.1 at 600 nm. Plates with samples were incubated over night at 37°C, then zones of inhibition were measured in tenths of a millimeter and coated suture samples were transferred to newly inoculated Agar plates. This process was repeated twice for two days. A more detailed description of the zones of inhibition assay is given in literature [43].

### Scanning electron microscopy (SEM) for structural analysis of coated sutures

In order to inspect quality of suture coatings as well as integrity, SEM pictures were taken without bacterial exposure at lower magnifications (up to 200x) to achieve an overview perspective. For this purpose, novel antimicrobially coated sutures, as well as uncoated and commercially available suture samples were prepared for common SEM. During preparation of suture samples, gold was sputtered on the suture samples at 5 x 10^−2^ mbar two times for 40 sec each with a Bal-tec Med020 coating system (Bal-tec, Balzers, Liechtenstein). Hereby, a thin gold layer of approximately 28 nm was generated improving image quality by generating conductive surfaces and protecting biological objects [45]. Pictures were taken for this investigation using a low vacuum SEM type JSM 6060LV (JEOL, Freising, Germany). Regarding the thermally labile suture—consisting of PGA—a low acceleration voltage of 5 kV was chosen.

### Scanning electron microscopy (SEM) for visualization of adherent bacteria

Additionally, SEM inspections were executed at higher magnification (2,500x) to investigate bacteria adherence on coated suture samples after bacterial exposure. SEM investigations were performed after washing of inoculated sutures, and before sonication. The number of adhered bacteria was estimated by using the field of view from SEM pictures (approximately 50 x 50 μm^2^) and counting visible adhering bacteria. The mean of three pictures from three sutures was calculated. Semi-quantitative levels for adhered bacteria were defined (low: up to 50 bacteria, moderate: 50 to 200 bacteria, and high: > 200 bacteria). Suture samples exposed to bacteria were treated with 4% paraformaldehyde in 0.01 M pbs solution for at least 1 h. This fixation step stabilizes the biological structure of the attached bacteria by cross-linking of proteins [46] and simultaneously inactivating bacteria. Subsequently, bacteria-containing suture samples were dried, gold sputtered as described and investigated by SEM.

### Viability of adhered bacteria on coated sutures (bacterial adhesion assay)

To quantitatively investigate the influence of antiseptic suture coatings on the viability of adhered bacteria, coated and uncoated suture samples were inoculated in bacterial suspensions for 3 h at 37°C using *Staphylococcus aureus* (ATCC^®^49230^™^). Attached viable *S*. *aureus* numbers on suture samples were measured after sonication and incubation of detached bacteria. Viable bacteria were determined by growth on Mueller Hinton II Agar plates (MHA; BD Diagnostic Systems, Heidelberg, Germany) and counting of colony-forming units (cfu). The bacterial adhesion assay described by Gollwitzer et al. [18, 47] was modified using the following procedure:

Mueller Hinton Broth (MHB; BD Diagnostic Systems, Heidelberg, Germany) was used to cultivate bacteria in suspension. Bacterial concentration was adjusted with a biophotometer (Eppendorf, Hamburg, Germany) at a wavelength of 600 nm. Suture samples of 1 cm in length (n = 10) were put in 1.5 ml-tubes filled with 1 ml *S*. *aureus* suspension at an initial concentration of 1.3 x 10^8^ cfu/ml (OD600 = 0.1). The tubes were incubated in a thermo-shaker (Unimax 1010, Heidolph Instruments, Schwabach, Germany) for 3 h at 37°C while shaking at 200 rpm. To remove weakly adhered bacteria from sutures, a washing process involving dipping the sutures 3 times in 1 ml sterile isotonic saline (0.9%) was performed. Subsequently, to remove strongly adhered bacteria from suture surfaces, samples were put into tubes with 1 ml sterile 0.01 M phosphate buffered saline (pbs: NaCl 0.138 M, KCl 0.0027 M; pH 7.4; P3818, Sigma-Aldrich, Germany) and treated using a 3-step procedure: (1) vortexing for 10 sec, (2) sonication for 1 min using an ultrasound at 35 kHz/280W (Sonorex RK255H, Bandelin, Berlin, Germany), and (3) vortexing for 10 sec. The obtained bacterial suspension was diluted to 1:10, 1:100, 1:1,000, and 1:10,000 with sterile 0.01 M pbs, and 100 μl of each dilution were plated in double on MHA plates. After 24 h of incubation at 37°C, colony-forming units were counted and the number of viable adhered bacteria on the suture surfaces was calculated. The numbers obtained were compared to those obtained from the following references: uncoated PGA suture (Gunze), palmitic and lauric acid coatings (PA80, LA80), commercially available absorbable sutures (Vicryl® and PGA Resorba®) with fatty acid coating, and triclosan-coated sutures (Vicryl® Plus). For all determined numbers of viable adhered bacteria a logarithmic reduction was calculated referred to the uncoated Gunze suture (G). Significance tests were compared in general to uncoated Gunze (G) and especially for antimicrobially coated sutures to the commercial antimicrobial suture Vicryl® Plus (VP).

### Viable bacteria of suspensions after suture incubation

To investigate potential growth inhibition on suture-surrounding bacteria in planktonic form, antimicrobial sutures were incubated for 3 h in *S*. *aureus* suspensions followed by detecting viable numbers of bacteria. Therefore, suture samples at 1 cm length were incubated in *S*. *aureus* suspensions during the *viability adhesion assay* experiments as described above. The turbidity of the bacterial suspension was measured at 600 nm (OD600) using a biophotometer (Eppendorf, Hamburg, Germany) for each inoculated suture at the beginning and at the end of experiment after 3 h. Numbers of viable bacteria were determined via a calibration curve for the bacterial test strain. The number of viable planktonic bacteria was compared to the bacterial growth in the presence of uncoated Gunze suture (G) and a logarithmic reduction was calculated after 3 hours of incubation for each suture sample.

### Evaluation of results of 11 μg/cm drug-containing sutures in regard to former studies

To determine the best novel antimicrobial suture for medical need, we evaluated the 11 μg/cm drug-containing novel chlorhexidine- and octenidine-coated sutures (CL11, CP11, OL11, and OP11) in comparison to the antimicrobial control Vicryl® Plus (VP). Results of the present study as well as from former studies [43, 44] were taken into account for comparative evaluation. Thus, for each relevant aspect (viability of bacteria adhered or in suspension, numbers of bacteria detected via SEM, biocompatibility, drug release kinetics and efficacy in zone of inhibition tests), semi-quantitative levels were defined.

### Statistics

Mean values and standard deviations were calculated from at least five independent measurements. Student’s t-test was performed for testing on equality of data sets at significance levels p < 0.05 (*), p < 0.01 (**), and p < 0.001 (***). The distribution of data was checked for each group referred to the mentioned controls via F-Test in Microsoft Excel® 2013. These results were taken into account during the student’s t-test. The Gaussian error propagation law was used for the subsequent use of flawed values. GraphPad Prism 6.0 (GraphPad® Software, La Jolla, CA, USA) was used for data evaluation and visualization of the result graphs.

## Results

### Antimicrobial efficacy against multiple relevant pathogenic bacteria

**Fig 1:**
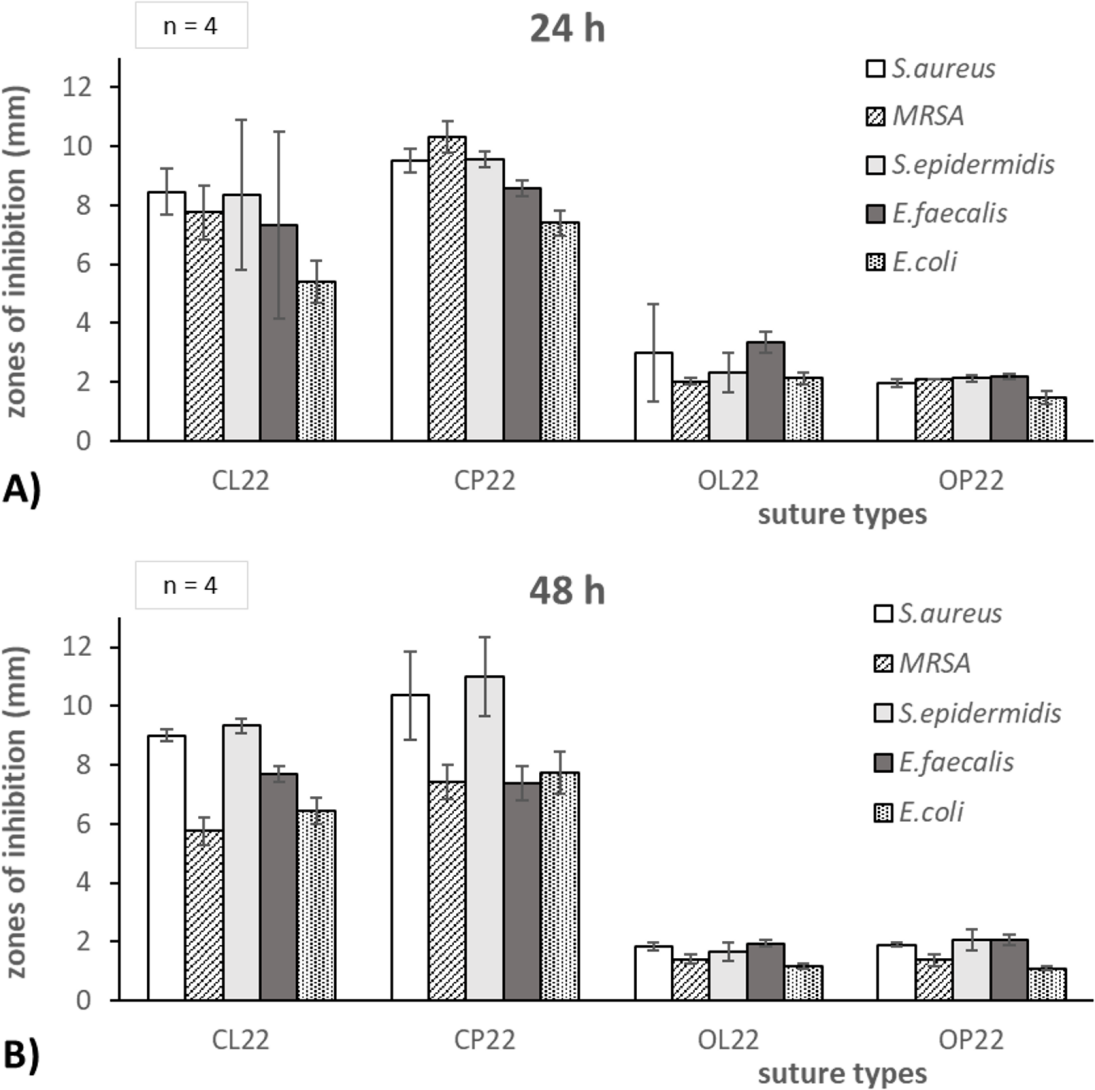
Zone of inhibition assay for five bacterial species over 48 hours. Zones of inhibition in millimeter for each coating type at 22 μg/cm drug content (CL22, CP22, OL22, OP22) on sutures. Test strains used were *S*. *aureus*, *MRSA*, *S*. *epidermidis*, *E*. *faecalis* and *E*. *coli* after A) 24 hours and B) 48 hours test period.

In general, high antimicrobial efficacy was found for all of the tested bacterial strains over the relevant test period of 48 hours (Fig 1). The type of coating affected the sizes of inhibition zones, especially the type of coated drug. On average, chlorhexidine-coated sutures inhibited bacteria at 8.3(±1.4) mm and octenidine-coated sutures at 2.3(±0.5) mm after 24 hours. After 48 hours, the inhibition zones were on average 8.2(±1.7) mm and 1.7(±0.4) mm for chlorhexidine and octenidine coatings, respectively. The antibacterial efficacy of novel coated sutures against tested bacterial strains was comparable to the most relevant bacterial strain *S*. *aureus*, used for further detailed investigations on bacterial adhesion.

### Scanning electron microscopy (SEM) for structural analysis of coated sutures

**Fig 2:**
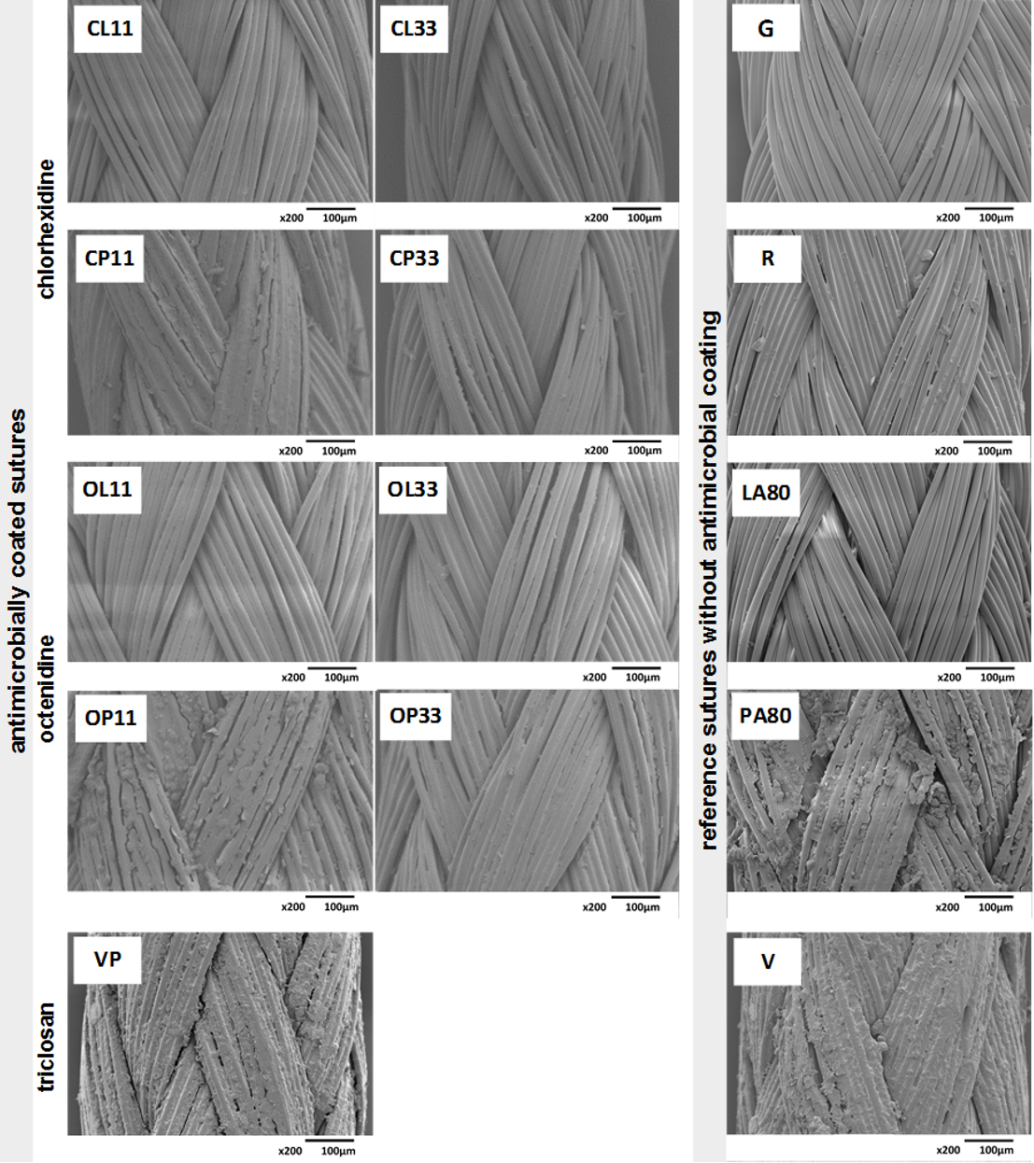
SEM pictures prior to bacterial exposure to inspect coating quality and suture integrity (magnification 200x). **Left**: Chlorhexidine- and octenidine-coated sutures for the lowest and highest drug concentrations used. Chlorhexidine sutures (CL11, CL33, CP11, and CP33) and octenidine-coated sutures (OL11, OL33, OP11, and OP33) are shown for laurate or palmitate carriers. Commercial antimicrobial sutures Vicryl® Plus (VP). **Right**: Reference sutures without antimicrobial drugs. Plain PGA suture material Gunze used for preparations (G) and commercially available resorbable sutures PGA Resorba® (R) and Vicryl® (V). Furthermore, sutures coated solely with fatty acid lauric acid (LA80), or palmitic acid (PA80) were investigated. Images are representative of three numbers of fields from three suture replicates.

Novel coated sutures show sustaining integrity and uniformly covered surfaces by drug-containing coating layers. There are hardly detectable differences via SEM for suture coatings using concentrations at 11 μg/cm, 22 μg/cm and 33 μg/cm. Therefore, sutures with the lowest and highest drug concentrations (11 μg/cm and 33 μg/cm) are presented (Fig 2, left). The reference suture (G) shows the structure of the uncoated suture material used for preparing antimicrobial sutures. Both drug carrier preparations (palmitic acid and lauric acid) completely covered the suture surface. The lauric acid-containing coatings CL11, OL11, CL33, and LA80 sutures showed smooth surface layers around each single filament. In contrast, a rougher structure of palmitic acid-containing coatings CP11, OP11, CP22, and PA80 was a characteristic feature. In general, the surface roughness of palmitate using novel coated sutures was comparable to commercially available sutures such as Vicryl® Plus, Vicryl®, and PGA Resorba® (Fig 2, right).

### Scanning electron microscopy (SEM) for visualization of adherent bacteria

**Fig 3:**
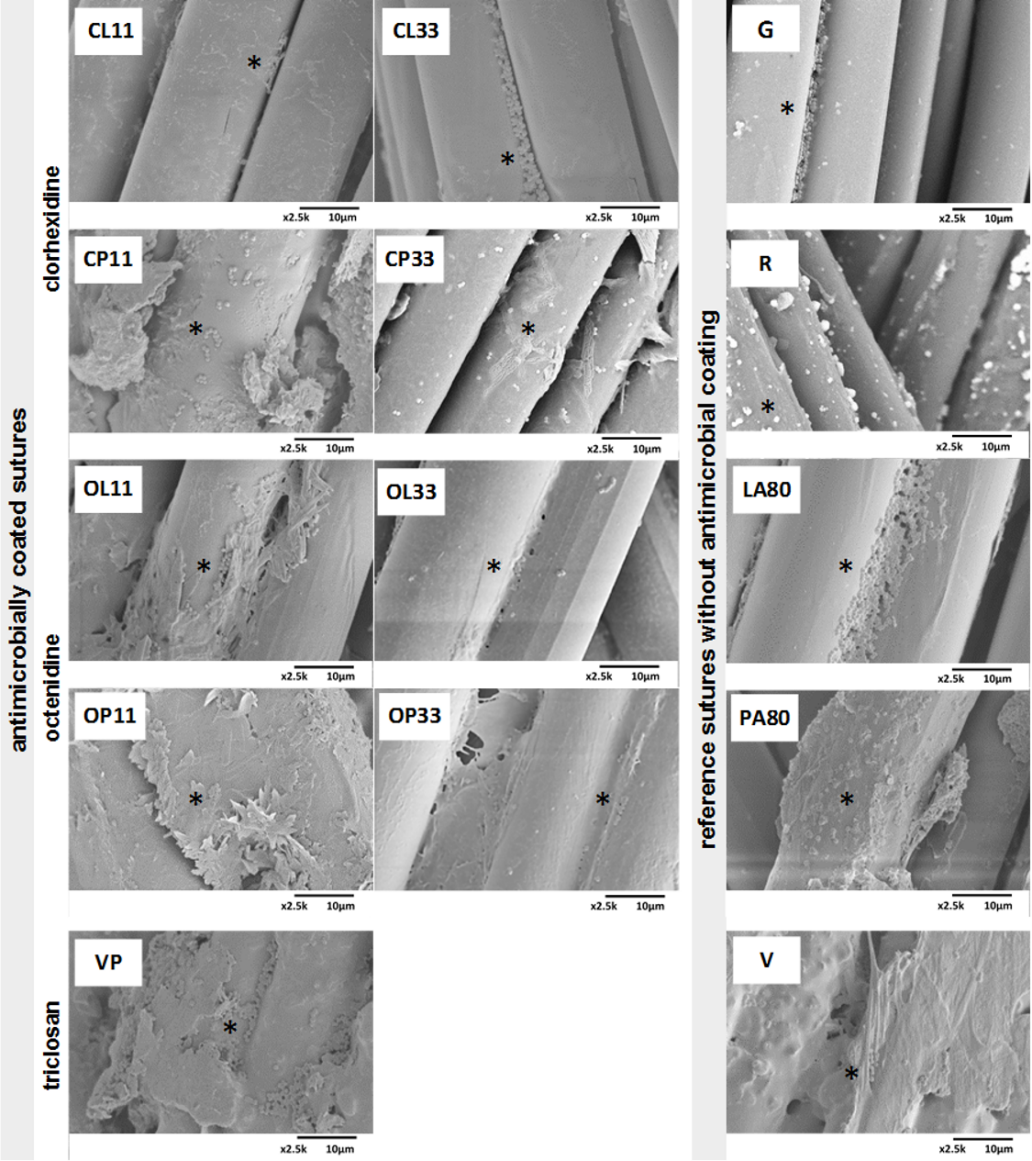
SEM pictures following bacterial exposure of coated sutures to visualize adhered bacteria and estimate their number semi-quantitatively (magnification 2,500x). Sutures were incubated in *S*.*aureus* suspension at 1.3 x 10^8^ cfu/ml for 3 hours. **Left**: Novel antimicrobially coated sutures are shown for the lowest and highest drug concentrations at 11μg/cm and 33μg/cm, respectively. Chlorhexidine-coated sutures (CL11, CL33, CP11, and CP33) and octenidine-coated sutures (OL11, OL33, OP11, and OP33) depicted for laurate or palmitate carriers. The commercial antimicrobial triclosan reference Vicryl® Plus (VP) is also shown in the last row. **Right**: Suture references without antimicrobial substances (G, R, LA80, PA80, and V). Adhered bacteria were exemplarily marked with an asterisk (*). Images are representative of three numbers of fields from three suture replicates.

In particular, the antimicrobial control (VP), the commercial triclosan-coated suture Vicryl® Plus showed relatively high numbers of adhering bacteria. All tested novel antiseptic coated sutures (CL11, CP11, OL11, and OP11) showed numerous bacteria on their surfaces (Fig 3: left), even for sutures at higher drug concentrations (CL33, CP33, OL33, and OP33). Numerous adhering bacteria were detectable on the non-antimicrobial suture control (G) and other sutures without antimicrobial substances (Fig 3: right; LA80, PA80, V, R, and G). Especially, inside gaps between single filaments, a high accumulation of *S*. *aureus* colonies was found on top of the fine coatings of lauric acid components as well as on the rough lumps of palmitic acid coatings.

### Viability of adhered bacteria on coated sutures (bacterial adhesion assay)

**Fig 4:**
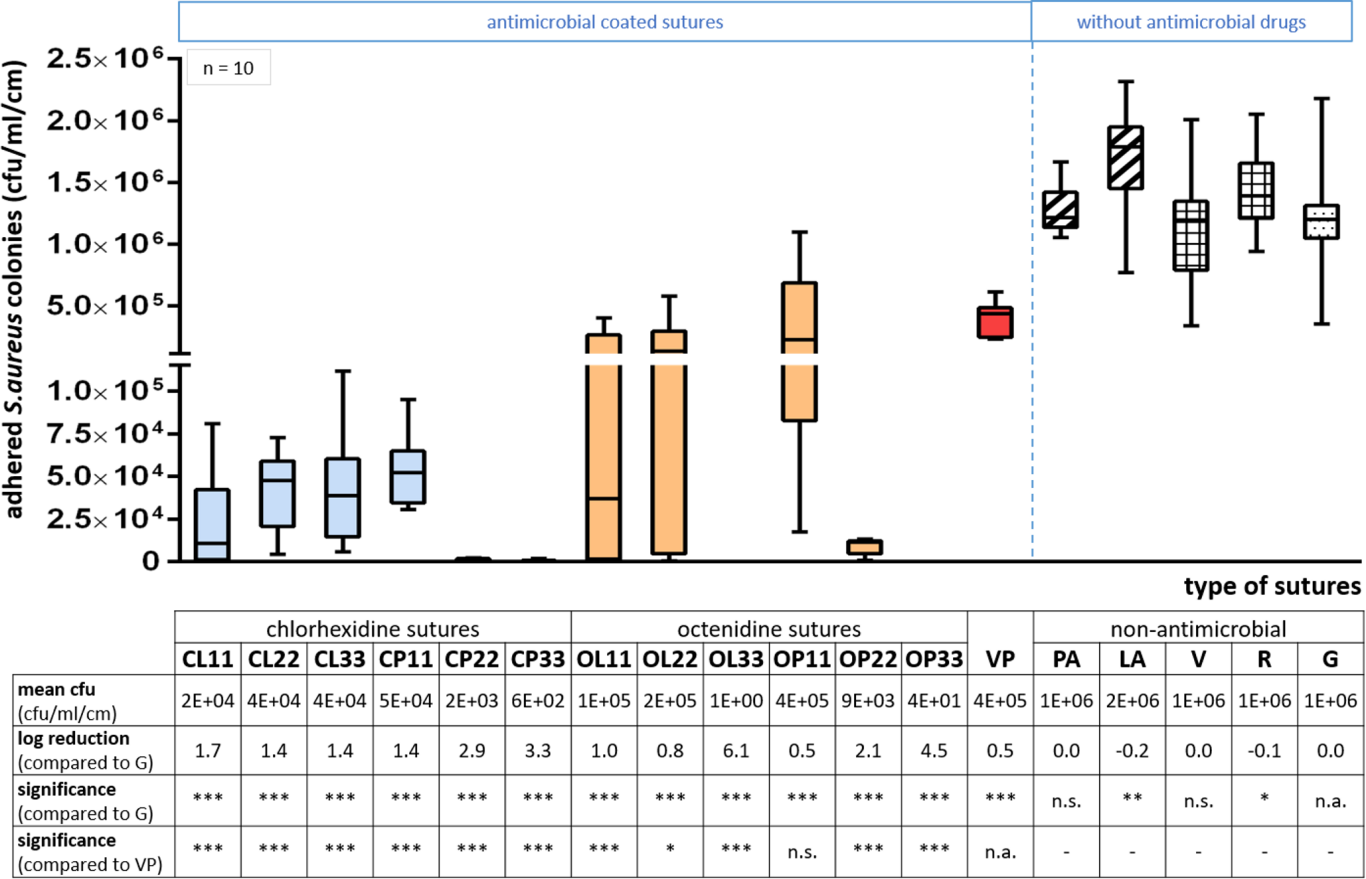
Numbers of adhered *S*. *aureus* colonies on sutures’ surfaces per cm sample after 3 hours of incubation in on average 1.3 x 10^8^ cfu/ml bacterial suspension. Viably adhered numbers of bacteria and their reductions compared to uncoated Gunze (G) suture. **Left** (up to dashed line): Sutures coated with antimicrobial substances, such as chlorhexidine-laurate (CL), chlorhexidine-palmitate (CP), octenidine-laurate (OL), and octenidine-palmitate (OP) each with the drug concentration 11, 22, and 33 μg/cm. Novel coated sutures were also compared to commercially available triclosan-containing Vicryl^®^ Plus (VP) suture. **Right**: Groups of sutures without active antimicrobial agents, uncoated Gunze (G), coated with fatty acids (PA80, LA80) and commercially available common resorbable sutures (V: Vicryl^®^, R: PGA Resorba^®^). Significance levels are p<0.05 (*), p<0.01 (**) and p<0.001 (***); n.s.: not significant, n.a.: not applicable.

Chlorhexidine-laurate suture (CL11) shows the lowest numbers of viable adhered bacteria within the 11 μg/cm drug-containing novel coated sutures. Compared to the antimicrobial control Vicryl® Plus, CL11 shows a 1.2 log greater reduction. In general, chlorhexidine and octenidine coatings exhibit lower colony numbers of viable adhered *S*. *aureus*, as compared to the non-antimicrobial control (G). The number of viable adhered bacteria of each novel antimicrobially coated suture type (CL, CP, OL, OP) and Vicryl® Plus (VP) was statistically significantly reduced (p < 0.001: ***; Fig 4) compared to sutures without active substances (PA80, LA80, V, R, and G). In comparison to the triclosan-containing suture (VP), representing the antimicrobial suture control, the novel sutures showed an even more significant reduction of viable adhered bacteria (p < 0.001: ***; CL11-CL33, CP11-CP33, OL11, OL33, OP22, and OP33) and (p < 0.05: *; OL22). Adhered bacteria were slightly inhibited by OP11 (n.s.), comparable to Vicryl® Plus. The reduction of viable adhered bacteria (Fig 4) was expressed by a logarithm of the basis 10, calculated for each tested suture. Bacterial log reductions were calculated referred to uncoated Gunze suture (G) without any drug. Significance tests were referred to the control (G), and on the other hand to triclosan-coated Vicryl® Plus suture (VP). Chlorhexidine- and octenidine-coated sutures demonstrated a high log10 reduction of adhered *S*. *aureus* colonies in the range of 0.5 (OP11) up to 6.1 (OL33) compared to uncoated Gunze suture (G). In contrast, triclosan-containing Vicryl® Plus suture demonstrated a small 0.5 log reduction against adhering bacteria.

### Viable bacteria in suspensions after suture incubation

**Fig 5:**
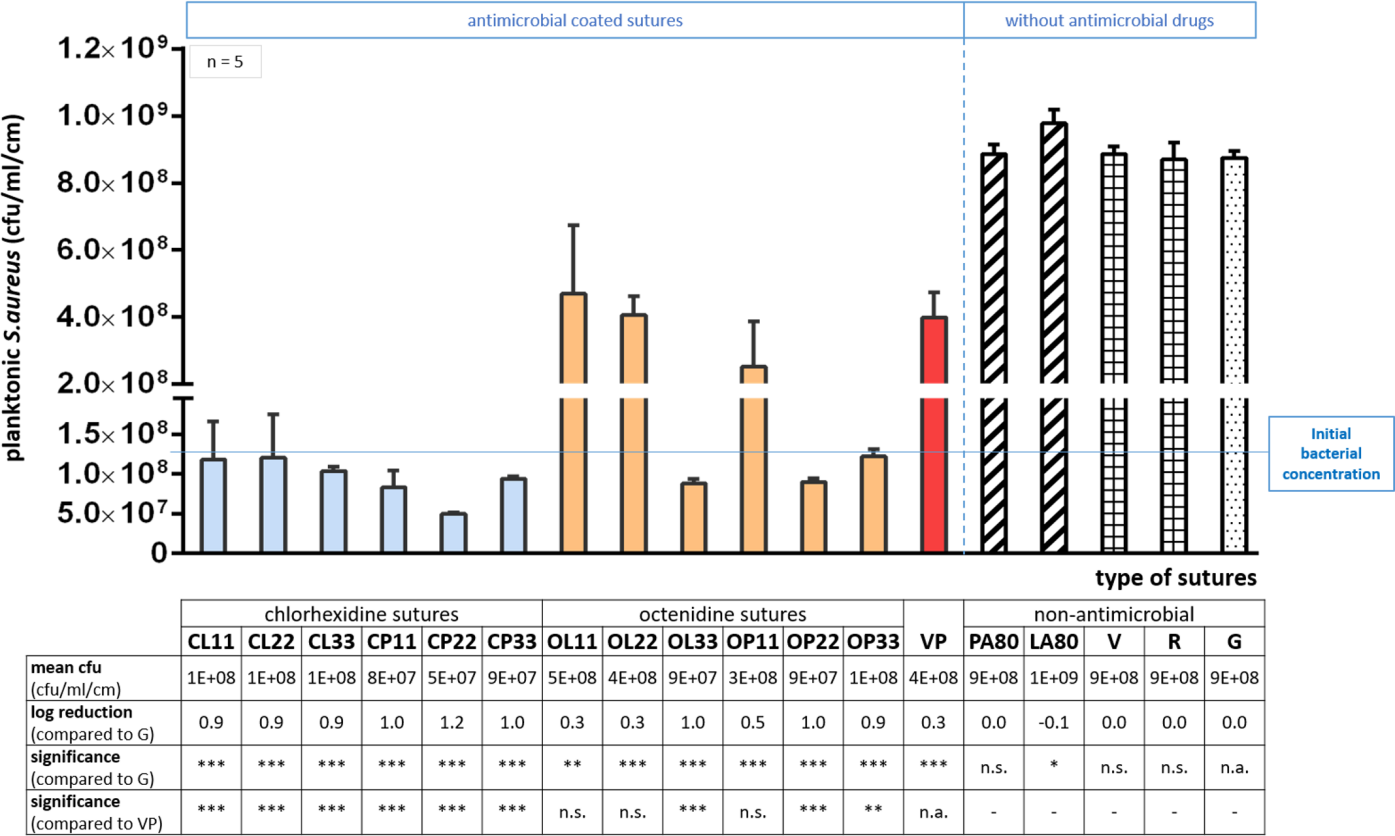
Numbers of viable bacteria in suspension incubated for 3 hours with novel antimicrobial sutures. An initial *S*. *aureus* concentration of 1.3 x 10^8^ cfu/ml was used for bacterial suspensions. Chlorhexidine- or octenidine-coated sutures showed a strong inhibition of pathogens in the surrounding suspensions. The triclosan-coated suture Vicryl^®^ Plus (VP) and the uncoated Gunze suture (G) were used as controls. Fatty acid-coated sutures (PA80, LA80) and commercial sutures without any drug content (V: Vicryl^®^, R: PGA Resorba^®^) were tested within the non-antimicrobial suture group. Significance levels are p<0.05 (*), p<0.01 (**) and p<0.001 (***); n.s.: not significant, n.a.: not applicable.

The reduction of planktonic bacteria in suspensions within the 11 μg/cm drug-containing novel antimicrobial sutures is lowest for the chlorhexidine-palmitate (CP11) and laurate sutures (CL11). Compared to the antimicrobial control Vicryl® Plus, CP11 and CL11 showed a greater bacterial reduction of 0.7 log and 0.6 log, respectively. In general, suspension bacteria were highly inhibited by the novel bactericidal sutures (Fig 5), whether coated with chlorhexidine or octenidine for each used concentration. Bacterial reductions were referred to the non-antimicrobial suture control (G), showing similar unaltered bacterial growth in suspensions similar to other tested sutures without antimicrobial substances (PA80, LA80, V, and R). In comparison to uncoated Gunze (G), Vicryl® Plus (VP) showed a bacterial reduction of 0.3 log, comparable to OL11 and OL22. Tested chlorhexidine-coated sutures decreased bacteria in suspension even further (p < 0.001: ***; CL11-CL33 and CP11-CP33) with at least 0.9 log. In addition, most of the octenidine sutures also showed a higher reduction of suspension bacteria compared to (G): OP11 (0.5 log), OL33 (1.0 log), OP22 (1.0 log), and OP33 (0.9 log).

### Evaluation of results of 11 μg/cm drug-containing sutures in regard to former studies

The chlorhexidine-laurate sutures (CL11) best met medical requirements. CL11 shows the lowest number of viable bacteria on sutures, a high drug release within the first 48 h, as well as good biocompatibility. Potentially, each of the four novel coated sutures using 11 μg/cm drug concentration can be clinically applied, since they are antimicrobial effective over several days and biocompatible [43, 44]. The experimental data from the current study (Table 2: white background) and earlier studies [43, 44] (Table 2: blue, orange and light gray background) were compared to each other. The rating levels (+, ++ and +++) used for the comparison of antimicrobial sutures are declared in Table 3.

**Table 2:** Evaluation of the novel antimicrobial sutures using chlorhexidine or octenidine at 11 μg/cm drug compared to commercial triclosan-containing Vicryl^®^ Plus.

Type of antimicrobial sutures	Viability of bacteria		SEM investigation	Biocompatibility	Delayed drug release	Zones of inhibition	
	log reduction of adhered bacteria	log reduction of planktonic bacteria	number of adhered bacteria	metabolic activity (%)	residual content (%)	duration (d)	initial size (mm)
**CL11** [43]	+++	+++	+	+	+	++	++
**CP11** [43]	+++	+++	++	++	+++	++	++
**OL11** [44]	+++	+	++	++	+	+++	+
**OP11** [44]	+	+	++	+++	+++	+++	+
**Vicryl**^**®**^** Plus** [43, 44]	+	+	+++	+++	n. d. ^a^	+++	+++

^a^ No determination of drug release, because of triclosan’s extremely low solubility in aqueous media. Referred to other *in vitro* studies by Ming et al. [48] and Edmiston et al. [18], the triclosan release was rated as +++ level.

Data from our recent study (white background) concerning reduction of viable adhered, as well as bacteria in suspension, and SEM investigations were arranged next to each other. Additionally, data from earlier studies [43, 44] are considered for evaluation regarding cytotoxicity, antimicrobial efficacy via zone of inhibition assay over time, and the slow drug release properties (dark blue background: chlorhexidine-sutures, orange background: octenidine-sutures, and light gray background: Vicryl^®^ Plus suture control).

**Table 3:** Rating levels used for comparative antimicrobial suture evaluation.

Sutures properties that are compared		Rating levels		
		+	++	+++
**Viability of bacteria**	log reduction of bacteria	≥ 0.3	≥ 0.6	≥ 0.9
**SEM investigation**	number of adhered bacteria	< 50	50–200	> 200
**Biocompatibility**	metabolic activity	≥ 60%	≥ 70%	≥ 80%
**Delayed drug release**	residual content after 96 h	≥ 10%	≥ 40%	≥ 60%
**Zones of inhibition**	initial size after 24 h	≥ 1 mm	≥ 4 mm	≥ 10 mm
	days of duration	≥ 1 d	≥ 4 d	≥ 8 d

The recent data demonstrated that chlorhexidine-laurate suture (CL11) shows the most efficient inhibition of adhered bacteria, which is critical for local infections. Therefore, we recommend the CL11 suture compared to Vicryl® Plus as an optimal surgical supplement to reduce wound infections. Nevertheless, octenidine-containing sutures at 11 μg/cm can also be helpful in applications where a slower and longer lasting drug release should be necessary.

## Discussion

In this study, we found that novel antimicrobial sutures using chlorhexidine or octenidine coatings were effective against multiple bacterial pathogens. Especially, viable adhering and surrounding planktonic *S*. *aureus* were strongly inhibited. Additionally, we found that reduction of adherent bacteria via novel sutures could be up to 12-fold higher than achievable with commercial antimicrobial suture Vicryl® Plus using triclosan. Scanning electron microscopy (SEM) pictures were used to investigate suture coatings and to demonstrate bacterial adhesion. The main finding of the present study was that novel antimicrobially coated sutures show considerably less viable bacteria on suture surfaces than triclosan-containing suture Vicryl® Plus. Therefore, these novel coated sutures may reduce suture-associated surgical site infection (SSI) more effectively than otherwise possible today. SSI is still an issue in medical daily routine. Sutures can promote such infections via the so-called “wicking effect” as well as by enabling bacteria to colonize. Sutures themselves affect bacterial adhesion, especially due to the chemical composition of suture material, surface structure, as well as capillarity. It has been shown that the property of sutures acts as a substrate for adhering bacteria can be correlated with the rate of infection [9]. Viable adhering bacteria form biofilms on suture surfaces by proliferation. These biofilms are even detectable on sutures in “culture-negative” SSI, a special form of wound infection in which no bacterial pathogens could be cultured using conventional diagnostic methods. [11] Antimicrobial-coated sutures also inhibit adhering bacteria and can be an established adjunctive aspect in reducing SSI [15] and thus interrupt this infection pathway.

The zone of inhibition assay showed a multispecies efficacy of novel coated sutures against the five tested relevant bacterial species. Therefore, a potential inhibition of clinical relevant pathogens is assumed. The efficacy is mainly dependent on the type of drug used for coating (chlorhexidine or octenidine) but also–to minor degree–on the drug carrier (laurate or palmitate). Overall, the antibacterial efficacy of coated sutures was comparably to the clinically most relevant *S*. *aureus* species. Therefore, *S*. *aureus* was used to investigate the bacterial adhesion in further detail. The inhibition zones indicated a sustaining broad-activity over the tested 48 hours. The amount of drug release is directly indicated by the size of inhibition zones. The suture‘s drug release persists for more than two days. That “fits” well with the description in literature [43, 44] antimicrobial efficacy lasting for nine days using octenidine coatings and for up to five days with chlorhexidine coatings. Novel coated suture materials protected broadly against microbes for the critical period of 48 hours after surgery, which is necessary to avoid SSI. Moreover, there is a high local efficacy against problematic MRSA infections.

Structural investigations by SEM of coated sutures showed uniformly distributed antimicrobial coatings on surfaces around the multifilament structure. Dependent on the type of fatty acid carrier, there was a detectable difference concerning the level of roughness. For lauric acid coatings, the fine structure of suture filaments was preserved. Coatings containing palmitic acid seemed to laminate filament strands resulting in a high degree of roughness, probably an effect of the presence of longer hydrocarbon chains. This observation is comparable to commercial sutures, such as Vicryl®, PGA Resorba®, and Vicryl® Plus. Especially, absorbable braided sutures are using coatings consisting of calcium stearate formulations to improve handling [49] and to smoothen the surface. Thus, the tissue damage by braided sutures during suturing, the so-called “sawing action” is reduced [50]. Calcium stearates consist of stearic acid, a fatty acid with an 18-carbon chain that is comparable to palmitic acid.

SEM pictures of inoculated sutures with 1.0 x 10^8^ cfu/ml *S*. *aureus* suspension over 3 h indicated numerous adhering bacteria on all suture surfaces whether coated with antimicrobial agents or not. Bacterial adherence seems to be independent from substance, drug carrier, surface roughness, or drug concentration. Especially, the gaps between single filaments of uncoated or laurate-containing sutures represent areas which were colonized by bacteria leading to a pearl chain arrangement (Fig 3, e.g. Gunze, CL33 and LA80). Adhering bacteria on surgical sutures represent a potential risk for wound infections and need effective inactivation to counteract infections. The sutures’ capillarity acts as a door opener for pathogens to penetrate into wounds as these microorganisms may trigger infection [51].

Some authors detected biofilm formation of bacteria grown on suture surfaces [52]. Further SEM pictures of inoculated suture samples also potentially demonstrated the production of little extracellular matrix around adhered bacteria, indicating the beginning of biofilm formation on sutures. Adhered bacteria were detached by sonication and viable bacteria were quantified afterwards. A strong inhibition of initially adhered bacteria during a short period of incubation was detected for the novel antimicrobial sutures. Therefore, an inhibiting effect on biofilm formation on sutures can be strongly expected. Sutures using lauric acid showed a higher number of adhering bacteria than those using the palmitic acid carriers. This conspicuousness was confirmed by the bacterial adhesion assay, proving higher numbers of viable adhering bacteria for lauric acid containing sutures.

The bacterial adhesion assay indicated a drastic log reduction of viable adhered bacteria on novel antimicrobially coated sutures. Compared to the weak log reduction of Vicryl® Plus the bacterial inhibition by contact with the novel antimicrobial sutures can be up to 12-times higher dependent on the kind of substance, drug carrier, and drug concentration employed. We suggest that the adhesion of bacteria could not be avoided by numbers via antimicrobial coatings. However, antimicrobial agents inside novel coatings significantly reduced the number of viable adhering bacteria in our experiments. Novel coated sutures may inhibit bacterial proliferation on suture surfaces and thus inhibit the initial biofilm formation. Consequently, novel chlorhexidine- and octenidine-coated sutures may have a higher ability to prevent SSI related to suture material than Vicryl® Plus. This effect could be limited by the numbers of microbes inside the incision or on the threads, and the sensitivity of bacteria against the type of drugs used in the coating layers. Sutures coated with fatty acid carriers only showed a slightly higher number of viable adhered bacteria compared to Gunze sutures without any coating (G). Especially lauric acid coatings (LA80) seem to attract adhering bacteria more than palmitic acid suture (PA80). Thus, laurates are presumably more suitable as drug carriers than palmitates to achieve low bacterial adherence.

Regarding the ultrasound treatment to release adhered bacteria, sonication is a competitive process between releasing and killing adhering bacteria, dependent on the duration of sonication [53]. Therefore, a short sonication time of 1 min was chosen, resulting in a low killing rate versus a detectable viable bacterial release. In combination with vortexing an increased soft release could be achieved [54]. Since all sutures were treated equally, sonication and vortexing is a meaningful process to release bacteria. Antimicrobial coatings are not able to reduce bacterial adhesion in general. However, they are an effective method to inactivate viable adhering bacteria [47].

Bacterial growth was investigated by incubation of *S*. *aureus* suspensions including coated and uncoated suture samples. Planktonic bacteria were highly inhibited by chlorhexidine- or octenidine-coated sutures compared to sutures without antimicrobial coating. In comparison to the commercially available triclosan suture Vicryl® Plus, a much higher effectiveness of bacterial reduction was demonstrated for all types of tested chlorhexidine sutures, as well as for the higher concentrated octenidine sutures. In addition, lower concentrations of octenidine in sutures showed a similar inhibition effect to that obtained by Vicryl® Plus. The efficacy was strongly dependent on the drug type, presumably in regard to its individual solubility and therefore differing drug release from suture coatings. Solubility in aqueous media was extremely low for triclosan, higher for octenidine and highest for chlorhexidine. We hypothesize that free drug molecules combined with a certain drug concentration would be necessary for an effective antimicrobial activity against bacteria.

Siedenbiedel and Tiller described multiple mechanism for antimicrobial surfaces, the killing effect on surrounding pathogens by drug releasing surfaces and the direct contact inactivation on surfaces, as well as a repelling effect on microorganisms [55]. We hypothesize that antimicrobial sutures based on fatty acid drug carriers inhibit surrounding pathogens by drug release, and on the other hand inhibit viable pathogens by direct contact with drug molecules during attachment on surfaces. We also presume that bacterial inhibition is dominated by surface inactivation or rather bactericidal effects on surfaces, due to the highly reduced number of viable adhered bacteria.

The present study has some important limitations: Although, the broad antibacterial efficacy of novel coated sutures has been shown in a zone of inhibition assay, only the clinically most relevant pathogen *Staphylococcus aureus* (*S*. *aureus* ATTC®49230™) was used for the bacterial adhesion assay. Staphylococci species are the most common pathogens responsible for wound infections and a variety of implant-associated infection [40, 56]. However, to identify the full potential of this approach, further research has to investigate bacterial adhesion by using other relevant strains. Nevertheless, the high multispecies antimicrobial efficacy via inhibition zones presumably also indicates high degrees of inactivation of other adhering bacteria. Our monospecies microbiological setup for investigation of bacterial adhesion is able to provide answers to the potential effect on initially adhered staphylococci and thus results in the potential to inhibit the following biofilm formation. Data was collected advantageous without any interference by interactions between different species. A further limitation is the sonication itself as a competitive process between detachment of bacteria and potential harm. Due to methodological constraints, the absolute number of bacteria adhering to suture surfaces could not be detected. The counts of cfu from surface-released bacteria only represent the viable content of adhering bacteria. Moreover, when using SEM pictures for visualization of adhering bacteria, it is not possible to distinguish between viable and inactivated bacteria. The fluorescence microscopy technique combined with a live/dead bacterial staining assay could solve this problem in future studies.

In summary, the zone of inhibition assay documented a bacterial multispecies efficacy over 48 hours. SEM investigations showed uniformly covered suture surfaces by coating and different roughness dependent on the type of fatty acid carrier. Furthermore, adhering *S*. *aureus* were found on each kind of tested suture, whether coated with antimicrobial substances or not. The number of viable *S*. *aureus* adhering on suture control groups was extremely high, without any drug and on the antimicrobial control Vicryl® Plus. Therefore, coated sutures presumably could not avoid bacterial adhesion itself. At the same time, novel coated sutures using chlorhexidine or octenidine inhibited adhered *S*. *aureus* significantly. Chlorhexidine-laurate suture (CL11) shows the lowest remaining number of viable adhered bacteria, despite an extremely high concentration of *S*. *aureus* inoculation. These bacteria are critical for the onset of local infections, thus this suture has the highest potential to further reduce the rates of SSI. Consequently, CL11 best fulfills the medical need and we recommend this suture type compared to Vicryl® Plus as an optimal surgical supplement to reduce wound infections. Furthermore, planktonic bacteria in suspension were also drastically inhibited by the novel coated sutures chlorhexidine-laurate, chlorhexidine-palmitate and octenidine-palmitate at 11 μg/cm. Octenidine-laurate at 11 μg/cm exceptionally showed a similar number of adhered bacteria to Vicryl® Plus, which nevertheless represents a reduction of 0.5 log compared to uncoated Gunze sutures. Octenidine-containing sutures at 11 μg/cm (OL11) can also be helpful in applications where a longer-lasting drug release may be necessary, e.g. when infections already exist during septic surgery.

Novel coated sutures in former studies showed excellently adjustable antimicrobial efficacy and release kinetics, lasting some days for chlorhexidine formulations and up to nine days for octenidine coatings. Dependent on the kind of drug, it could therefore possibly be useful to distinguish between two fields of application: on the one hand, a long-term drug release, e.g. for wound closure during septic surgery. On the other hand, applications with a shorter drug release, e.g. for infection prophylaxis in common surgery.

The present study fundamentally demonstrated a much higher inactivation of viable adhering bacteria through novel antimicrobially coated sutures and thus, presumably, a much higher potential to interrupt the”wicking effect” compared to Vicryl® Plus. Therefore, we suppose that the novel sutures have a higher potential to avoid suture-associated SSI. Pre-clinical studies, followed by clinical investigations are necessary to demonstrate their ability to avoid SSI in vivo and prove their safety. Novel antimicrobial sutures using chlorhexidine or octenidine at 11 μg/cm drug content may pose an alternative in case of triclosan resistance, or to extend the active substances clinically used on antimicrobial sutures. This should give surgeons an additional effective tool to react to complex pathogen milieus and resistances.

## Conclusions

In this study, we found that the novel chlorhexidine- and octenidine-coated sutures are effective against multiple bacterial species over the critical period of 48 hours after surgery. The analysis in detail for *S*. *aureus* revealed that antimicrobial sutures at 11 μg/cm drug content demonstrate superior bactericidal properties against adhering *S*. *aureus* compared to commercial triclosan-containing Vicryl® Plus. Especially, the chlorhexidine-laurate coating (CL11) shows the highest efficacy to minimize the number of adhered as well as planktonic bacteria. This coating provides a high drug release in the first, clinically most relevant 48 h after suture application and is–in addition–highly biocompatible. Therefore, this coating type best meets the medical needs and should be recommended for potential clinical application. The high reduction of viable adhering bacteria on this novel coated suture is a promising approach to improve prevention of surgical site infections in routine surgery. These results encourage further pre-clinical and clinical trials to confirm safety and efficacy of this coating technology *in vivo*.
