# Correction: Viable adhered *Staphylococcus aureus* highly reduced on novel antimicrobial sutures using chlorhexidine and octenidine to avoid surgical site infection (SSI)

**DOI:** 10.1371/journal.pone.0193284

**Published:** 2018-02-15

**Authors:** Andreas Obermeier, Jochen Schneider, Norbert Harrasser, Jutta Tübel, Heinrich Mühlhofer, Dominik Pförringer, Constantin von Deimling, Peter Foehr, Barbara Kiefel, Christina Krämer, Axel Stemberger, Matthias Schieker, Rainer Burgkart, Rüdiger von Eisenhart-Rothe

There is an error in affiliation 4 for author Matthias Schieker. Affiliation 4 should be: Klinik für Allgemeine-, Unfall- und Wiederherstellungschirurgie, Klinikum der Universität München, München, Germany.

## References

[1] Obermeier A, Schneider J, Harrasser N, Tübel J, Mühlhofer H, Pförringer D, et al (2018) Viable adhered *Staphylococcus aureus* highly reduced on novel antimicrobial sutures using chlorhexidine and octenidine to avoid surgical site infection (SSI). PLoS ONE 13(1): e0190912 https://doi.org/10.1371/journal.pone.0190912 29315313 10.1371/journal.pone.0190912 PMC5760023

